# Current Evidence of Natural Agents in Oral and Periodontal Health

**DOI:** 10.3390/nu12020585

**Published:** 2020-02-24

**Authors:** Gaetano Isola

**Affiliations:** Department of General Surgery and Surgical-Medical Specialties, Unit of Oral Surgery and Periodontology, School of Dentistry, University of Catania, Via S. Sofia 78, 95124 Catania, Italy; gaetano.isola@unict.it

**Keywords:** periodontitis, natural agents, gingivitis, antioxidants, vitamins

## Abstract

Oral and periodontal diseases, chewing disorders, and many destructive inflammatory diseases of the supporting tissues of the teeth are usually caused by an imbalance between host defense and environmental factors like smoking, poor nutrition, and a high percentage of periodontopathogenic bacteria. For these reasons, it is important also to focus attention on plaque control and also on improving host resistance through smoking and stress reduction, and a healthy diet. During the last decades, the importance of micronutrients has been extensively reviewed, and it was concluded that the prevention and treatment of periodontitis should include correct daily nutrition and a correct balance between antioxidants, probiotics, natural agents, vitamin D, and calcium. Recently, there has been growing interest in the literature on the impact of nutraceutical dietary aliments on oral and general health. This Special Issue provides a current and thoughtful perspective on the relationship of diet and natural agents on oral and periodontal diseases through a correct clinical approach with the last and most important evidence that may determine good oral conditions and high quality of life.

It has been widely demonstrated that herbal medicines, which include medicinal herbs, herbal preparations, and phytotherapeutic compounds (that have plant or natural materials), have real therapeutic benefits for humans [1,2,3]. Worldwide, about 80% of the population uses phytotherapeutic products such as extracts, vitamins, tea, and other similar principles for various reasons for the treatment of various pathologies, with a cost of over 50 billion dollars a year in the global market [4]. This high consumption of herbal products compared to traditional drugs, such as antibiotics, is attributable to the large margin of safety and tolerability of natural agents, which could lead to a possible reduction in the long-term on the total national economic costs compared to traditional drugs. In addition, conventional drugs have also been shown to have a higher incidence of side effects, allergies, and resistance, especially antibiotics [5,6]. Therefore, herbal medicines are increasingly being used both as food supplements and to prevent or treat common oral and systemic diseases [5].

Among the main diseases of the stomatognathic apparatus, periodontitis is a chronic inflammatory disease caused by oral bacteria that determines the destruction of the supporting structures of the teeth [7,8,9]. The etiology of periodontitis is multifactorial with the bacteria of the oral biofilm which are fundamental for the initiation and progression of the disease. The different forms of periodontal disease are very different around the world but reach a total incidence rate of over 60%. The bacterial origin of periodontitis has been widely demonstrated, starting from an imbalance in aerobic and anaerobic biofilm bacteria [10], which can lead, under specific conditions, to activation of the host response, especially of neutrophilic bacteria and related products, which determines the disruption of soft and hard oral tissues [11,12,13]. This imbalance of the host response through the immune system results in further up or down-regulation of various pro-inflammatory cytokines, which finally determines the release of rapid oxidative stress (ROS) cells and neutrophil mediators [14,15]. This prolonged inflammatory status on the hard and soft tissues of the periodontium, including the connective tissue, leads to the degradation and consequent loss of the periodontal structure of the tooth and of the alveolar bone, causing, in the final disease steps, tooth loss [16].

Several studies have shown, in damaged periodontal tissues, a direct association between increased levels of inflammatory mediators induced by reactive oxygen species (such as NO) and the worsening of periodontitis [17,18]. Therefore, herbal medicines have been demonstrated to have an important role due to their broad spectrum of action against ROS and NO mediators, together with a good safety and tolerability margin compared with traditional drugs in both children and adults [19,20,21,22,23].

A good adjuvant response in both surgical and nonsurgical periodontal treatment has been shown in recent years by natural agents. Especially in the non-surgical approach, various antimicrobials and chemotherapy agents, including chlorhexidine, triclosan, desiccant agents, vitamin and probiotic compounds, and cetylpyridinium chloride, have been studied and validated for the management of periodontitis [24,25,26,27,28,29]. However, even more studies have aimed at analyzing phytotherapeutic drugs in order to obtain antimicrobial, antiseptic, anti-inflammatory, and antioxidant effects during periodontitis.

In fact, herbal medicines have been shown to possess a wide and specific range of biological properties including antimicrobial, antioxidant and anti-inflammatory effects at the oral and systemic levels. The natural phytotherapeutic compounds, including medicinal herbs, help to suppress the inflammatory response, which determines, in the long term, the destruction of the hard and soft tissues of the oral cavity, characteristic in various oral diseases, including periodontitis [30,31,32,33,34]. Among the main anti-inflammatory actions due to phytotherapy drugs, there is, above all, an anti-inflammatory and oxidative action which leads to excellent therapeutic action in the long-term. However, on the other hand, various studies in the oral field that have analyzed the actions of traditional and phytotherapeutic drugs have given uncertain results that require large-scale populations to be validated [35,36,37,38,39].

Based on these findings, the aim of this Special Issue is to further analyze the therapeutic effects of these medicinal herbs, phytotherapy, and of the main inflammatory mediator characteristics of oral and periodontal diseases.

Given the many new aspects related to the optimal management of phytotherapy drugs in dentistry, it was my pleasure to receive publications detailing the results of different joint research groups for this highly stimulating Special Issue on this subject that is aimed at analyzing and validating new scientific approaches to improve the prevention and treatment of oral and periodontal diseases through the use of phytotherapeutic drugs.
